# Supplementary material for: Medium-Chain Fatty Acids Rescue Motor Function and Neuromuscular Junction Degeneration in a Drosophila Model of Amyotrophic Lateral Sclerosis
Source: Cells. 2023 Aug 28;12(17):2163. doi: 10.3390/cells12172163 (PMC10486503; doi:10.3390/cells12172163)
Supplement: Supplementary file 1 [file cells-12-02163-s001.zip › cells-2551275-supplementary.pdf]

Supplemental Figures

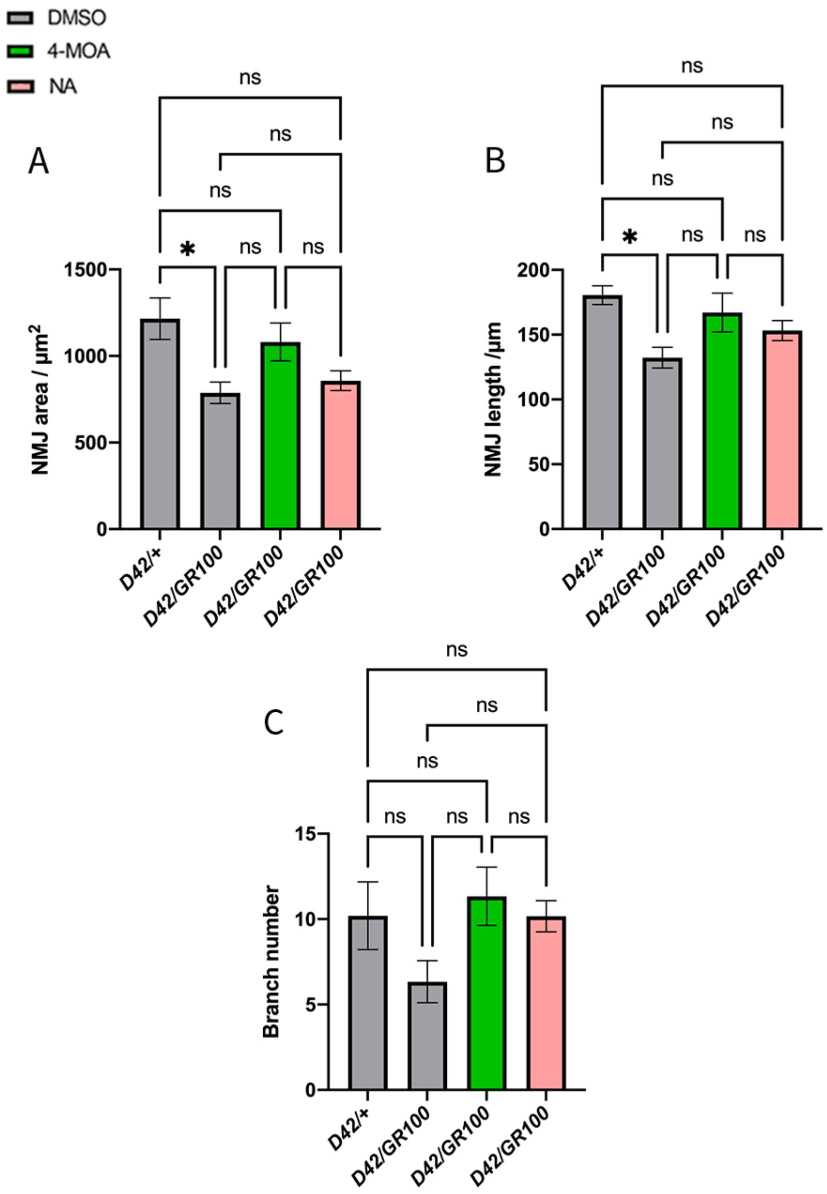

Supplemental Figure S1: GR100 expression reduces NMJ length and area, but does not affect NMJ branching. Quantification of NMJ area (A) NMJ length (B) and branch number (C) in indicated genotypes, and with 4-MOA (green) and NA (pink) treatment (*n*=5-6). Controls were given equal volumes of DMSO (grey) (n.s. = not significant).

Supplemental Tables

Supplemental Table S1. Association of metabolites with ALS pathology and MCFA treatment in whole larvae.

	D42/+ Vs. D42/GR100		D42/GR100 Vs D42/GR100 (4-MOA)		D42/GR100 Vs D42/GR100 (NA)	
	**p-value**	**FC[a]**	**p-value**	**FC[b]**	**p-value**	**FC[b]**
Urea	0.027	0.63	0.023	1.40	0.014	1.29
Alanine	0.004	0.64	0.003	1.84	0.001	1.92
GABA	0.002	0.62	2.79×10^{-5}	1.99	0.002	2.71
Cytosine	0.446	1.15	0.033	0.66	3.12×10^{-4}	0.49
Uracil	0.123	1.31	0.622	0.94	3.96×10^{-4}	0.39
Nicotinamide	3.38×10^{-5}	3.76	4.98×10^{-5}	0.34	2.73×10^{-4}	0.40
Nicotinate	4.52×10^{-5}	0.44	2.49×10^{-4}	2.45	0.001	1.92
Asparagine	1.42×10^{-4}	2.26	0.024	0.75	0.006	0.72
Ornithine	0.071	0.74	0.022	1.57	0.395	0.88
Aspartate	0.005	8.54	0.008	0.21	0.008	0.19
Glutamate	0.001	3.89	0.008	0.51	0.002	0.35
Arginine	0.010	1.69	0.407	0.90	0.016	0.64
Citrulline	0.006	2.29	0.079	0.66	0.019	0.56
Cytidine	0.001	2.62	0.001	0.40	2.99×10^{-4}	0.18
Uridine	0.041	0.47	0.026	2.04	3.17×10^{-4}	2.27

[a] fold change calculated relative to D42/+, [b] fold change calculated relative to D42/GR100 untreated. Abbreviation: FC; fold change, GABA; γ-aminobutyrate.

Supplemental Table S2. Association of metabolites with ALS pathology and MCFA treatment in the CNS.

	**D42/+ Vs. D42/GR100**		**D42/GR100 Vs D42/GR100 (4-MOA)**		**D42/GR100 Vs D42/GR100 (NA)**	
	**p-value**	**FC[a]**	**p-value**	**FC[b]**	**p-value**	**FC[b]**
Urea	0.155	0.76	0.557	0.86	0.517	1.03
Alanine	Not measured					
GABA	0.028	0.61	0.087	1.55	0.894	1.59
Cytosine	0.144	2.82	0.432	1.43	0.120	0.58
Uracil	Not measured					
Nicotinamide	0.012	0.62	0.380	1.12	0.960	1.13
Nicotinate	0.013	0.53	0.037	1.39	0.001	0.72
Asparagine	Not measured					
Ornithine	Not measured					
Aspartate	0.492	0.74	0.156	1.98	0.210	3.05
Glutamate	0.021	0.26	0.031	3.27	0.681	3.88
Arginine	0.048	0.66	0.940	1.01	0.616	1.15
Citrulline	Not measured					
Cytidine	0.053	0.43	0.617	0.89	0.327	2.48
Uridine	Not measured					

[a] fold change calculated relative to D42/+, [b] fold change calculated relative to D42/GR100 untreated. Abbreviation: CNS; central nervous system, FC; fold change, GABA; γ-aminobutyrate.

Supplemental Table S3. Association of metabolites with ALS pathology and MCFA treatment in the muscle.

	**D42/+ Vs. D42/GR100**		**D42/GR100 Vs D42/GR100 (4-MOA)**		**D42/GR100 Vs D42/GR100 (NA)**	
	**p-value**	**FC[a]**	**p-value**	**FC[b]**	**p-value**	**FC[b]**
Urea	0.208	0.68	0.396	0.69	0.640	0.84
Alanine	0.088	2.59	0.891	0.96	0.330	0.64
GABA	0.271	0.72	Not measured		0.849	0.94
Cytosine	0.020	0.59	0.014	2.22	0.571	1.20
Uracil	0.313	1.33	0.036	0.34	Not measured	
Nicotinamide	0.035	0.20	0.180	0.70	0.465	0.83
Nicotinate	0.004	0.60	1.70×10^{-4}	2.05	0.954	0.99
Asparagine	0.277	0.69	0.192	1.55	0.897	0.95
Ornithine	0.025	0.61	0.741	0.94	0.040	0.53
Aspartate	0.165	0.69	0.114	1.45	0.004	1.92
Glutamate	6.74×10^{-5}	0.23	0.809	0.91	0.472	1.26
Arginine	0.002	0.56	0.872	0.97	0.100	0.72
Citrulline	0.011	0.32	0.151	0.40	0.073	0.18
Cytidine	0.002	0.46	0.028	0.66	0.086	0.65
Uridine	0.008	0.45	0.538	3.30	0.179	0.68

[a] fold change calculated relative to D42/+, [b] fold change calculated relative to D42/GR100 untreated. Abbreviation: FC; fold change, GABA; γ-aminobutyrate.

Supplemental Table S4: Association of metabolites with ALS pathology and MCFA treatment in the gut.

	D42/+ Vs. D42/GR100		D42/GR100 Vs D42/GR100 (4-MOA)		D42/GR100 Vs D42/GR100 (NA)	
	**p-value**	**FC[a]**	**p-value**	**FC[b]**	**p-value**	**FC[b]**
Urea	not measured					
Alanine	not measured					
GABA	not measured					
Cytosine	0.651	0.73	0.781	0.86	0.867	1.10
Uracil	0.337	1.27	0.065	0.68	0.513	0.85
Nicotinamide	0.301	1.41	0.425	0.78	0.063	0.42
Nicotinate	0.141	0.68	0.001	2.20	0.016	2.04
Asparagine	0.328	0.70	0.876	0.93	0.835	1.10
Ornithine	0.810	1.14	0.378	0.53	0.329	0.47
Aspartate	0.140	0.67	0.582	0.84	0.512	1.44
Glutamate	0.125	0.53	0.586	0.75	0.451	1.88
Arginine	0.195	1.33	0.687	0.89	0.638	1.15
Citrulline	0.284	0.74	0.978	1.01	0.899	1.04
Cytidine	0.806	0.96	0.283	0.79	0.466	0.80
Uridine	0.159	0.85	0.004	0.50	0.161	0.69

[a] fold change calculated relative to D42/+, [b] fold change calculated relative to D42/GR100 untreated. Abbreviation: CNS; central nervous system, FC; fold change, GABA; γ-aminobutyrate.
